# Task-specific modulation of memory for object features in natural scenes

**DOI:** 10.2478/v10053-008-0037-9

**Published:** 2008-12-04

**Authors:** Alan Robinson, Jochen Triesch

**Affiliations:** 1 Department of Cognitive Science, University of California, San Diego,USA; 2 Frankfurt Institute for Advanced Studies, Johann Wolfgang Goethe University, Frankfurt, Germany

**Keywords:** visual working memory, natural scenes, natural tasks, change detection

## Abstract

The influence of visual tasks on short and long-term memory for visual features was investigated using a change-detection paradigm. Subjects completed 2 tasks: (a) describing objects in natural images, reporting a specific property of each object when a crosshair appeared above it, and (b) viewing a modified version of each scene, and detecting which of the previously described objects had changed. When tested over short delays (seconds), no task effects were found. Over longer delays (minutes) we found the describing task influenced what types of changes were detected in a variety of explicit and incidental memory experiments. Furthermore, we found surprisingly high performance in the incidental memory experiment, suggesting that simple tasks are sufficient to instill long-lasting visual memories.

## Introduction

During everyday life people are engaged in many tasks which rely heavily on visual information about the environment around them. Different tasks have different informational demands: Finding a sticky-note on your desk requires searching for a yellow rectangle among desk clutter, whereas writing on the same note requires details about the orientation of the paper’s surface and the location of your pen. Given the wide range of details which can be extracted about an object, it has been argued that people cannot process them all equally. Instead, they preferentially process the details of an object which are relevant to their current task (Hayhoe, 2000). Eye tracking in block-pattern copying experiments has revealed that task relevant blocks often receive multiple fixations, and the order of the fixations suggest that just one aspect, such as the color, or the location of an object is acquired during a single fixation (Ballard, Hayhoe, & Pelz, 1995). Neurophysiology work in monkeys has shown that task-relevant stimuli produce much stronger responses in many visual areas of the brain, such as LIP (Kusunoki, Gottlieb, & Goldberg, 2000) and V4 (Connor, Gallant, Preddie, & Van Essen, 1996). Even in early visual areas, such as V1, the firing rates of neurons are modulated by both visual input and the monkey’s current task (Crist, Li, & Gilbert, 2001). Task-dependent modulation of neural firing seems to be present at all levels in the visual system.

In this paper we investigate if the greater degree of processing for task-relevant features results in a more long-lasting representation of those features in visual memory for natural objects. We call this hypothesis “the task-relevant memory advantage”. It predicts that the features of objects which are relevant to a person’s current task will be more accurately recalled later. We hypothesize that this is driven by two mechanisms: (a) visual memory encodes the features of objects at different levels of strength^1^, and (b) the strength is modulated by how much those features are related to the subject’s current task. In this work we measure the task-relevant memory advantage in a variety of human psychophysical paradigms which also allow us to investigate the capacity of visual memory for natural scenes, and the extent that visual memories are formed purely by the act of engaging in simple tasks. Understanding how memories are formed during tasks is particularly important, since our every day life is predominantly driven by tasks. If the nature of each task influences what kinds of features are encoded, then the representations (both long and short term) that people form about their environment will reflect the tasks they engage in.

Most work on visual memory for natural scenes has focused on what objects are encoded, but not which aspects of those objects. Rensink, O’Regan, and Clark (1997) showed that people repeatedly missed large changes to natural scenes when made between 80 ms grey masks. Some changes, however, were noticed more easily than others (those designated as “central interest objects” in a norming study where subjects wrote a short sentence describing the scene), suggesting that some aspects of a scene are significantly more likely to be encoded in memory. Eye-tracking studies using the same stimuli (O’Regan, Deubel, Clark, & Rensink, 2000) showed that subjects were more likely to look at central interest items, which raises the possibility that the advantage for detecting changes to these items is due to a bias to fixate them. Interestingly, looking at a changing item did not always lead to change detection. Even when subjects looked directly at the location of change, 40% of the time they did not notice when a change occurred during a blink. This suggests that subjects did not encode everything near fixation. What was being encoded, however, is difficult to infer from their data. The authors suggest that subjects, while often attending to the objects near the center of gaze, were sometimes attending to objects outside the center of gaze instead. A plausible alternative interpretation of the data, however, is that at any given moment subjects were only processing certain features of the objects at the center of gaze and changes were only detected when those features changed.

The change detection literature has lead to some debate about the visual memory capacity for natural scenes. The Rensink et al. (1997) study is sometimes cited as evidence that visual memory is very limited, because people have such difficulty detecting changes (e.g., Noe, Pessoa, & Thompson, 2000). However, change blindness could be due to other factors, such as failures in the comparison process (Hollingworth, 2003; Simons & Rensink, 2005). Indeed, later work has shown that some aspects of natural scene memory can be surprisingly better than suggested by earlier change detection literature. Hollingworth (2004) showed that visual memory for the objects in computer rendered indoor scenes is quite accurate if subjects are forced to attend to the objects in a controlled order, and asked to detect changes to the previously attended objects in a Two-Alternative Forced Choice (2AFC) paradigm. Visual short-term memory (the last one or two items attended) was nearly perfect, and visual long-term memory for scenes (and many of the objects within them), though less accurate, persisted for several minutes. While subjects did forget many of the objects they fixated, they nonetheless remembered specific visual details about hundreds of recently fixated objects. How complete, however, were the representations subjects formed? It is possible that when people encode an object in visual memory they are able to accurately represent all of its features. On the other hand, in most of Hollingworth’s work, subjects only had to search for a single type of change (object replacement), which may have limited the types of features subjects needed to encode. Visual memory for all types of features may not have been equally long-lasting. Converging evidence for this comes from the work of Tatler, Gilchrist, and Rusted (2003). Subjects freely viewed natural scenes, and then were tested immediately in a 4AFC paradigm on either the presence of items in the scene, their location, color, or shape. It was found that the longer subjects viewed the scenes, the better their memory for all of these features, however the rate of improvement varied for different features; for instance color memory did not improve much after 5 s of viewing, whereas shape memory was still improving significantly even after 10 s. This suggests that features were encoded at different levels of strength initially, and with additional viewing time the encoding strength of those features could be improved.

In our prior work we also found evidence for selective processing of different features of objects (Triesch, Ballard, Hayhoe, & Sullivan, 2003; Droll, Hayhoe, Triesch, & Sullivan, 2005). Subjects were given the task of sorting colored blocks onto different conveyor belts based on their visual features in a Virtual Reality (VR) simulation. On a small percentage of trials, a change was made to a block while the subject was moving it. Even though the changed block was at the center of attention, many of the changes were missed, even when subjects were explicitly instructed to monitor for changes. Changes were least likely to be missed, however, when they were to the task-relevant features of the block. This suggests that task-relevant features of individual objects can receive preferential encoding in visual working memory. This result is somewhat surprising in light of the proposal made by Luck and Vogel (1997) that visual working memory can accurately store three or four objects, independent of the number of features of those objects. If the number of features does not matter, why would people only encode some features, or encode those features at different levels of strength? Our result could be made compatible with Luck and Vogel’s view if it is assumed that engaging in an active task prevents people from using their memory to its fullest capacity. Recently, however, other studies very similar to Luck and Vogel’s have found that the visual complexity of the objects decreases the number of objects that can be accurately maintained (Wheeler & Treisman, 2002; Delvenne & Bruyer, 2004; see also Alvarez & Cavanagh, 2004, for converging evidence from a different paradigm). If the number of features that can be represented accurately is limited, then it is sensible that our visual memory system minimizes the encoding of task-irrelevant features.

Other researchers have also used VR to explore how interacting with an environment can influence visual memory and change detection. Wallis and Bülthoff (2000) had subjects drive or passively observe a VR driving simulator. They found subjects were more likely to detect changes to items near the road, and that this effect increased when subjects had to drive the simulator, rather than just passively observing a pre-recorded route. While this provides further evidence that a task can influence what objects people encode in memory, it does not speak to whether the encoding of specific features is influenced as well. Similarly, Dornhoefer, Unema, and Velichkovsky (2002) had subjects pretend they were driving a car while viewing static pictures of roads and found that change detection was better for driving related changes (pedestrians and vehicles) than for driving unrelated changes (trees and signs). This suggests that a task can influence which objects in natural scenes are likely to be encoded in visual memory. This was not the central question of interest to their research, however, and thus they did not control for low-level saliency effects or include a condition were subjects had to pretend to engage in a non-driving task. It is therefore, at best weak evidence of task-effects in natural scenes, and also does not speak to whether the encoding of different features is influenced in natural tasks.

In the present work we ask if the task-relevant visual memory advantage that we found in VR also applies to pictures of natural scenes, specifically the same images used by Rensink and O’Regan in the studies described earlier. There are important differences between prior change detection research with natural scenes and our virtual reality experiments. The VR world is interactive and full of movement, but is visually simple and looks almost the same between trials, whereas the research with natural scenes uses highly complex, but static stimuli, and entirely new images are shown on each trial. Because of these differences, subjects show different visual memory capacities for objects in these experiments. Specific details of many objects in natural scenes can be remembered quite accurately over the period of several minutes. A specific configuration of blocks in our VR experiments, however, is likely to be lost as soon as a new trial with a new configuration of blocks begins. Thus, the task effects found in the VR experiments may be due to the greater difficulty subjects have remembering the stimuli.

In the following experiments we will explore how a simple describing task influences performance at detecting changes to different types of features of objects in natural scenes. In the first experiment we will show that when tested immediately after describing a scene, no task effects are found. In three following experiments we show that when tested over longer delays, task effects are found under a variety of conditions.

## Experiment 1: Active viewing task and immediate memory test

This experiment tests if a simple task - describing a single feature (color, identity [name], or location) of several objects within a scene - would selectively improve people’s memory^2^ for the feature described. Memory for the scenes was tested immediately after subjects described each scene by asking subjects to locate a change made to that scene. According to the task-relevant memory advantage hypothesis, subjects who describe the color of objects should perform better at detecting color changes, subjects who name objects should be better at detecting the addition or removal of objects from the scene, and subjects who describe the location of objects in the scene should be better at detecting that an object had moved within the scene. A baseline condition with no task was also included to compare the effect of having an active task versus just memorizing the scene.

The describing task controlled which objects subjects attended to and the order that they attended to them. This allowed us to test two additional questions: (a) To what extent do recently attended objects have a more detailed representation in memory? (b) Does Rensink’s central versus marginal object distinction derive exclusively from the increased likelihood that subjects will fixate those objects?

### Method

#### Stimulus

Sixty college students participated in the experiment (15 per condition). In this experiment, and all that follow, subjects were replaced if they were colorblind or had seen any of the stimuli before.

#### Apparatus

In this experiment and all that follow, stimuli were presented on a 15” LCD display at an average viewing distance of 0.6 m (subjects were free to move their head). The experiment was programmed using Matlab and the PC Psychophysics Toolbox (Brainard, 1997; Pelli, 1997).

#### Materials

We used 48 pictures of indoor and outdoor natural scenes taken from Rensink et al. (1997). In this stimulus set each original scene was paired with a changed version generated by photo manipulation software. Either an object’s color was changed (*color change*), its location was changed (*translation change*), or an object was removed or added to the scene (*object change*). Sixteen changes of each type were included. Half of the changes were made to objects rated as “central interest” based on the criteria that subjects frequently mentioned these objects when instructed to describe the pre-change version of these scenes in an independent norming study. The other half of changes were made to “marginal interest” objects, so designated because subjects did not mention them in the norming study. The low-level salience of the changes (number of different pixels, luminance, and color differences) were made roughly equal across central and marginal interest items. Full details of the construction of these stimuli can be found in Rensink et al. (1997). We also generated 14 practice stimuli which had the same manipulations as the Rensink stimuli, but with more obvious changes so that subjects could quickly learn what was expected of them.

Eight objects were selected for subjects to describe in each scene, equally distributed between central interest and marginal interest objects as long as a sufficient number of objects appeared in the scene (in a few scenes there were only three central interest objects, in which case five of the objects selected would be of marginal interest). Crosshairs were placed on top of these objects during the experiment. One of the eight crosshairs was on top of the object that would change in the scene (or on top of the location where an object would appear), centered on a location where the pixels would change between images. If the change in the scene involved an object moving (translation change), the crosshair on top of that object would also move; otherwise the placement of crosshairs was identical between the original images and their changed counterparts.

#### Procedure

**Figure 1.**
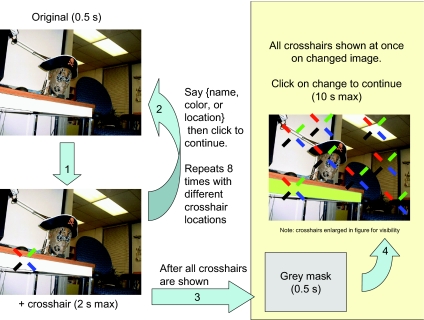
Example trial from Experiment 1.

Subjects had two tasks (see Figure 1). They completed both tasks for a single scene before moving on to the next one. First, they viewed a single version of a scene and described eight objects in the image. These objects were designated by displaying a crosshair over each object, one at a time, for up to 2 s. After describing the object, subjects would click the mouse button to advance to the next crosshair. If they did not click in time, the next crosshair would appear automatically. Before displaying each crosshair the scene was shown unobscured for 500 ms. Crosshairs were displayed in a random order between scenes and subjects, counterbalanced to uniformly distribute the length of the time intervals between describing the object that would change and searching for that change.

After all eight objects were described the scene was replaced with a grey mask for 500 ms, and then the subjects began the change detection task. The changed version of the scene was shown and all eight crosshairs for that image were superimposed on the screen at once. Subjects had 10 s to click on the crosshair that was on top of the changed object, and were told to guess if they were unsure.

Subjects were split into four between group conditions with different versions of the scene describing task: (a) name objects (*name task*), (b) say the dominant color of objects (*color task*), (c) say if the object was in the foreground or background of the scene (*location task*), or (d) a taskless control where subjects just memorized the objects under the crosshairs and did not have to describe anything. In the control condition subjects viewed each crosshair for 1.13 s, which was the average crosshair viewing time across subjects in the other three conditions. Each subject completed just one condition, but all subjects saw the same set of images, crosshair locations, and object changes.

### Results and discussion

#### Task effects

**Figure 2.**
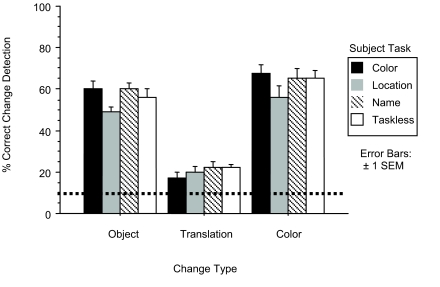
Accuracy on change detection task for Experiment 1. Dashed line represents chance performance.

We analyzed change detection accuracy using a repeated measures ANOVA in a three change type by three task analysis (the taskless condition was not included). There was no main effect of subject task, *F*(2,42) = 2.9, *p* < .06, though the p value was close to the standard .05 threshold for significance, suggesting that the type of task may have had an impact on overall accuracy rate for all change types. There was a strong main effect of change type, *F*(2,84) = 131, *p* < .0001, driven by the difficulty subjects had detecting translation changes. Indeed, subjects were only slightly above chance detecting translation in our paradigm (19% vs. chance at 12.5%). There was, however, no significant interaction effect, *F*(4,84) = 1.4, *p* < .25, suggesting that all change types were equally detectable across tasks (see Figure 2).

The lack of interaction suggests that the describing task had no effect on our subjects’ ability to recall different features of the scenes over short retention intervals. To assess this directly, we collapsed across color, location, and name tasks, and compared them to the taskless control condition. A repeated measures ANOVA found that the taskless condition was not reliably different from the collapsed conditions; main effect *F*(1,58) = .3, *p* > .5; interaction *F*(2,116) = .2, *p* > .8.

This analysis strongly suggests that the performance in the taskless control condition was equivalent to conditions where subjects described the objects in scenes. This implies that subjects can conduct the describing task and memorize the scene in parallel about as well as when just focusing on memorizing the scene. The describing task does not interfere with or enhance scene memory over the retention intervals tested in this experiment.

#### Order effects

Change detection performance did not depend on the number of intervening crosshairs between looking at the object that would change and conducting the change detection task. We quantified this with a linear regression analysis, which measured how well change detection performance could be predicted by how many additional crosshairs were displayed in a scene after a crosshair had been placed over the item that would change. The fit (*R* = .01) indicates no linear relationship between these variables. In addition, visual inspection of the data did not suggest that any higher-order relationships were present.

If subjects had been relying on short-term memory, we would have expected at least some change detection advantage for the most recently described items. The lack of order effects suggests that change detection performance was driven by long-term memory of the scene (see the general discussion section at the end of this paper for full consideration of what type of memory was tested). These representations appear to be fairly long-lasting, as once formed they did not fade significantly with time (subjects spent an average of 13.6 s describing each scene) or with interference from focused attention on up to seven other objects.

#### Item effects

**Figure 3.**
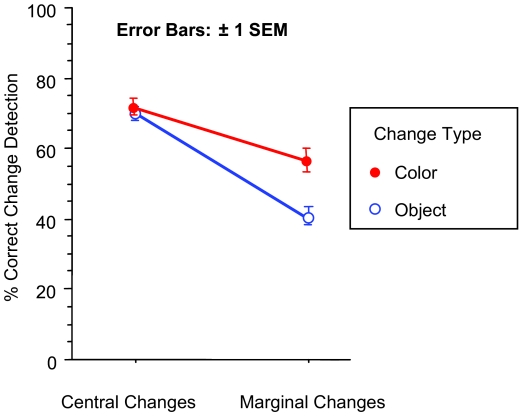
Accuracy on change detection task for Experiment 1. Dashed line represents chance performance.

Change detection performance was compared for “central” and “marginal” interest items in a repeated measures ANOVA with two factors: “interest” and change type (Figure 3). Translation changes were not included since performance was near chance for these items. In addition, we did not include the subjects who participated in the taskless condition, since the lack of task may have left extra time for subjects to make extra saccades during or between trials. There was a main effect of interest, *F*(1,44) = 75, *p* < .0001, with changes to central interest items detected more often than for marginal interest items. There was also a main effect of change type, *F*(1,44) = 7, *p* < .01, with color changes being easier to detect. In contrast to the central and marginal interest changes, the low-level salience of the color and object changes were not equated, so this result is likely due to how these stimuli were constructed, rather than a general advantage for remembering colors.

Finally, there was an interaction between factors, *F*(1,44) = 18, *p* < .0001, with color changes being less influenced by the “interest” manipulation.

### Conclusions

Previous eye tracking studies with the same stimuli have shown that subjects are more likely to saccade to central interest items (O’Regan et al., 2000). This raises the possibility that the change detection advantage for central interest objects within a scene may be reducible to what objects subjects’ saccade to, since changes to saccade targets are more likely to be noticed (Henderson & Hollingworth, 2003). O’Regan argues that a bias to saccade to central interest items does not explain the effect since subjects sometimes detect changes to objects outside of the fixation, suggesting that change detection is driven by the location of attention, not fixation. While this is a plausible argument, it doesn’t rule out that a fixation bias is causing the central item advantage.

In our data, however, subjects were always forced to attend to the object that would change, even when it was of marginal interest to the scene. While it is possible that subjects made additional fixations on the central interest items in the 500 ms gap between trials, our experience of running ourselves in pilot versions of this experiment suggests that this was exceedingly rare. Identifying and describing each object in 2 s or less was fairly demanding, and the 500 ms pause was just enough time to prepare for the onset of the next trial. In addition, we found that it felt very unnatural to look away from an object while describing it. Thus, fixation tendencies do not appear to explain the effect, or at least play a much smaller role than in free viewing paradigms such as O’Regan et al. (2000). Instead, it appears that memory for central interest items really is better. One possible contribution to this effect is that the central interest items play a more semantically important role in the scene; if they are added or removed it changes the interpretation of the scene. This semantic change serves as an additional cue for the target of the change. Indeed, object changes were much more likely to be detected for central than marginal interest items. This theory also explains why color changes are less impacted by the central versus marginal interest manipulation: Changing the color of these items rarely if ever changes the interpretation of the scene. Note, however, that there was a small improvement in color change detection rate for central interest items. This suggests an additional advantage for remembering central interest items which cannot be due to the color change influencing the semantic interpretation of the scene. Perhaps this is due to additional fixations on central interest items made in the gap between trials, though as discussed above we feel that these extra fixations would have been very rare.

We found no evidence for an interaction between the describing task and memory accuracy. It is somewhat surprising that describing precisely the feature of an object that will change confers no advantage for detecting that change only a few seconds later. It seems that the processing necessary to conduct the describing task did not influence the representations subjects formed of the scene, apparently refuting our hypothesis that the task-relevant features of objects are better encoded in memory.

On the other hand, the change detection task was performed almost concurrently with the describing task. Perhaps describing each object did not consume all of subjects’ attention, and subjects could also concurrently deploy attention to memorizing additional features of the object they were describing. Since subjects had many chances to practice change detection, they may have learned precisely what features to encode and maintain in memory to do well on the task, and this knowledge may have allowed them to diminish any effects the describing task would have otherwise had on how objects in the scene were encoded in memory. Indeed, Tatler et al. (2003) suggests that the amount of time people have to view a scene influences how many features of the objects in that scene are remembered, with more time leading to more features. Since completing the describing task took a while, subjects likely had enough time to also encode features they knew would be useful for the change detection task.

Another potential reason why the describing task had no impact on performance was that subjects were able to retain a great deal of information about the scenes over short periods of time. Irrelevant details for the describing task might have been encoded less strongly than task-relevant details, but perhaps over the retention period tested, either level of encoding was sufficient to maintain performance. Perhaps over a longer retention period, however, the differences in encoding strength would become more detectable. In Experiments 2-4 we will empirically test these two possibilities.

## Overview of Experiments 2, 3, and 4

In the following series of experiments we switch to testing longer-term memory, using a paradigm where the retention period is 1-4 min instead of just a few seconds). This allows us to test the effect of the describing task on memory under conditions of either explicit or incidental encoding. In Experiment 2, we show that task effects on memory do develop over longer retention intervals, by giving subjects a surprise memory test after all scenes are described. In Experiment 3, we show these task effects remain even when subjects know that their memory for the scenes they describe will be tested but do not know what sort of test or what features will be tested. In Experiment 4, we show that when subjects learn what features will change, the describing task still influences longer-term retention of those features, though less consistently than in Experiment 3.

## Experiment 2: Surprise memory test after describing scenes

The describing task should have maximal impact on what subjects remember about a scene when subjects are unaware that there is anything expected of them other than describing the scene. In addition, if differences in task-relevant and task-irrelevant encoding are difficult to detect over short retention periods, then with more delay these differences might become accentuated.

These two manipulations are naturally combined by giving subjects a surprise memory test after they have finished the describing task for all scenes. In this new paradigm, change detection performance will depend on features that are encoded in long-term visual memory as an automatic byproduct of the describing task.

### Method

#### Subjects

Forty four college students, all of whom were new subjects, participated in the experiment (22 per condition).

#### Stimuli

Five scenes with color changes and five scenes with object changes were selected from Experiment 1. To maximize power we selected only the scenes where subjects had detected changes 50% (± 5%) of the time. This criterion excluded all translation changes. Four additional images were selected for the subjects to practice the describing task; change detection was not conducted for these images.

#### Procedure

Subjects were told they were in an object naming experiment, and that their responses would be recorded. First they conducted the describing task for the four practice images and then the ten experimental images, using the same crosshair locations as in Experiment 1. In a between subjects manipulation, half described the color of objects (*color task*) and half named the objects (*name task*). The timing for the describing trials was identical to Experiment 1. Subjects described the versions of scenes that had been manipulated digitally, so that change detection trials would be conducted on images free of any manipulation artifacts. This was done to prevent subjects from searching for manipulation artifacts to aid in guessing which object changed. Since the object change trials were created by digitally removing an object from the scene, this meant that during change detection, the five object change trials always showed objects being added to the scene.

After describing all images, subjects were shown new instructions informing them that they were really in a memory experiment and explaining the change detection task. Their change detection performance was then tested on the ten experimental images, shown in the reverse order from when subjects had described them, so that a wide range of retention intervals would be tested (on average, as short as 55 s, and as long as 262 s, depending on whether the image was the last one described, or the first one, respectively). Since the experiment was self-paced, there was minor variability in the length of the retention intervals tested. Unlike Experiment 1, there was no time out for the change detection trials, since early piloting found that many subjects would otherwise timeout on the first few change detection trials. Instead, when 10 s were up, the computer beeped and subjects were verbally reminded to “just guess” if they had to. At the end of the experiment we administered a questionnaire to see if subjects had guessed that they should try to memorize the images they were naming during the first half of the experiment.

### Results and discussion

#### Timing

Subjects spent an average of 16 s per image during the describing task, 55 s reading the instructions for the surprise memory test, and then 7 s per image searching for changes. The average time spent on each task between subjects in the color condition and name condition differed by no more than 2%. The length of time spent inspecting each image to detect the change suggests that subjects conducted a serial search of the image, guided by the location of the crosshairs, and that change detection was not driven by bottom-up processes immediately drawing attention to the change location.

#### Task effects

**Figure 4.**
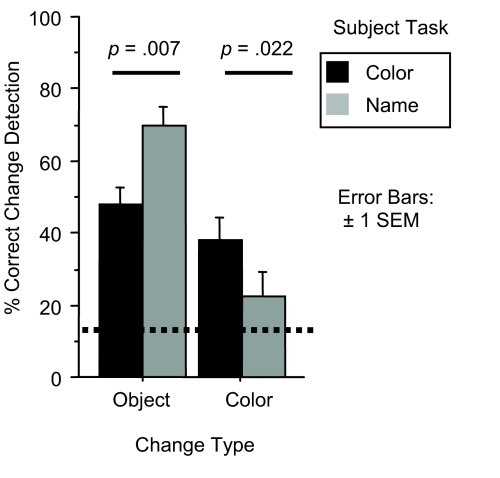
Performance on change detection task in Experiment 2. Dashed line represents chance performance.

In contrast to Experiment 1, the percent correct data collected in this experiment was markedly non-normal, probably due to the smaller number of scenes that each subject was tested on. For this reason we conducted planned comparisons between different conditions using the nonparametric Mann-Whitney U test (see Figure 4).

Subjects who completed the name task were at a relative disadvantage at detecting color changes, as compared to subjects who described the color of the objects (*U* = 164; *p* < .022). This suggests the subjects who named objects did not encode the color of the objects as strongly because it was of less relevance to their task.

Subjects who completed the color task were at a relative disadvantage at detecting object changes as compared to subjects who named the objects in the scene (*U* = 144, *p* < .007). This is somewhat remarkable, given that the color under the crosshair changed by virtue of the object under the crosshair changing. This suggests these subjects did not form as long-lasting a representation of the identity of the set of objects they described, even though they could later recall what color those objects were.

#### Questionnaire

At the end of the experiment we asked subjects to write the answer to two questions: “Before you started the experiment, did you think it would involve any memory tests?” and then “Did you make any effort to memorize what the pictures looked like?”. Out of 44 subjects, 42 gave an unequivocal no to both questions. For 2 subjects, the answers were more ambiguous; 1 subject in the object naming condition responded “Not really” to both questions, and 1 subject in the color condition responded “Yes” to the first and “No” to the second. This suggests that our cover story about studying object identification was convincing, and that most, if not all subjects, did not attempt to memorize the objects they viewed.

#### Overall accuracy

Given the long delay between describing the images and the memory test, and the fact that subjects were not expecting the memory test, we were surprised to find that subjects did so well at change detection. In Experiment 2, on average subjects correctly identified 59% of object changes, and 30% of color changes. In comparison, in Experiment 1 subjects detected 50% of the changes in the same images. Purely random guessing for these stimuli would lead to only 12.5% correct^3^, suggesting that subjects were remembering quite a lot of detail about the scenes.

### Conclusions

Our subjects exhibited long-lasting visual memory for the details of natural scenes. These memories were formed even though our subjects had no prior reason to remember the scenes. Furthermore, this experiment shows that a subject’s task can influence memory for particular features of natural scenes when subjects were not instructed to memorize anything. Note, however, that subjects were also able to detect changes that were not task relevant at an above-chance level, demonstrating that multiple features were encoded, including non-task relevant features. This suggests that different features of an object can be encoded with different levels of strength. The stronger the encoding, the more likely they are to recall the feature when tested later. Since subjects did not know they were in a memory experiment, this difference cannot be due to different levels of active rehearsal. Rather, it suggests that people’s default strategy while viewing natural scenes is to most strongly encode the details which are relevant to the person at that time. Since the describing task biased which details were important, it biased which features were best encoded in long-term memory. If, however, all details where known to be important (because of an upcoming memory test) perhaps people would be able to encode all features of an object with equal accuracy, even if they did not know specifically which features were important. Alternatively, the difference in performance could be due to the difference in delay, with task effects showing up only after greater delays.

## Experiment 3: Expected memory test after describing scenes

This experiment tests how much of the task effects in Experiment 2 were due to concealing the memory test from subjects until after all images were described. A secondary question is if overall memory performance would increase if subjects knew that remembering the scene would be useful. Finally, the presentation procedure was modified to facilitate direct comparisons of task-relevant and irrelevant recall rate over different retention intervals.

### Method

#### Observers

Forty four college students, all of whom were new subjects, participated in the experiment (22 per condition).

#### Stimuli

Same as Experiment 2.

#### Procedure

Identical to Experiment 2, except that before describing any images subjects were told they would be tested for subtle visual details of the scenes they described, and they should try to remember as much about the scenes as they could. After describing all images, the same instruction text as in Experiment 2 was presented to explain the exact procedure of the memory test.

In addition, we manipulated the random ordering of images to reduce variability between subject groups. Twenty two random orderings of scenes were created and each was used once for subjects completing the color task and once for the subjects completing the name task. In this way, between groups the same set of random stimuli orders were seen, so that any difference in performance would be due to subject variation and the subjects’ task, and not due to the order in which stimuli were presented.

### Results and discussion

#### Timing

On average, subjects spent 17 s describing each image, 47 s reading the change detection instructions after finishing the describing task, and 7 s per image searching for changes. The difference between average timings for the two tasks was at most 2%. Timing was also very similar to that of Experiment 2, except for reading the instructions between describing scenes and searching for changes, which subjects finished an average of 8 s quicker in this experiment.

#### Task effects

**Figure 5.**
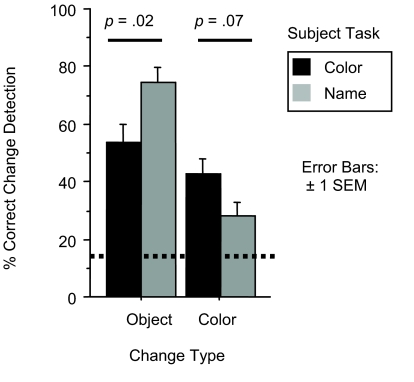
Performance on change detection task in Experiment 3. Dashed line represents chance performance.

The describing task influenced the features that subjects could recall about the originally described images (see Figure 5).

Though it was not quite statistically significant, subjects who completed the name task were at a relative disadvantage at detecting color changes, as compared to subjects who described the color of the objects (*U* = 169, *p* < .07). Subjects who completed the color task were at a relative disadvantage at detecting object changes as compared to subjects who named the objects in the scene (*U* = 143, *p* < .02).

#### Overall accuracy

**Figure 6.**
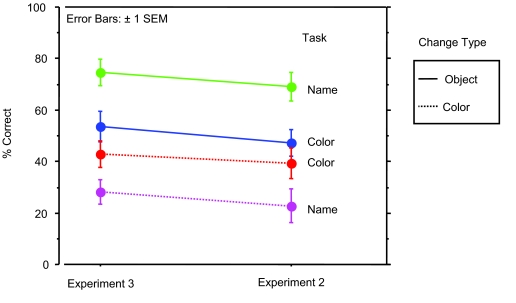
Comparing performance between Experiments 3 and 2.

In Experiment 3 accuracy increased over Experiment 2 by an average of 5% (see Figure 6). This increase, however, was not statistically reliable when we compared the average performance of subjects in Experiment 3 to the average performance of subjects in Experiment 2 (*U* = 803, *p* < .17). Thus, knowing that the experiment involved memory had at most a small effect and possibly no effect on how well subjects detected changes.

#### Order effects

**Figure 7.**
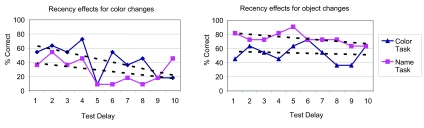
Percent correct change detection as a function of retention delay. Delay 1 corresponds to the image tested immediately after the change detection instructions are shown, and tested immediately after the instructions are read.

The task-relevant advantage was only found in Experiments 2 and 3, where the delay between describing images and change detection was much longer than in Experiment 1 (on average, 46 to 253 s longer, depending on the serial order of each image). Thus the length of delay may play an important role in measuring the task-relevant memory advantage. To explore this further, we analyzed how subjects’ change detection performance varied as a function of how long ago they described the stimulus (see Figure 7).

For color changes there is a trend of decreasing accuracy with increasing retention intervals, for both task relevant and irrelevant conditions (the best fit lines are y = -4.6x + 18%, and y = -2.4x + 15%, respectively; x represents retention interval), though the data is noisy. The task-relevant advantage exists for seven out of ten retention intervals, suggesting that task-relevance improved color recall for most of the duration of the experiment. Extrapolating from the best fit lines, however, it appears that the task-relevant advantage, as well as the majority of memory for object color would disappear if tested much beyond the longest intervals in this experiment.

For object changes the decrease in performance is less pronounced for both task relevant and irrelevant conditions (the best fit lines are y = -1.6x + 65%, and y = -0.5x + 51%, respectively). For eight out of ten retention intervals, subjects who named the objects were more accurate than subjects who described object color. The task-relevant advantage appears to be present for the duration of the experiment, and perhaps would extend over even longer delays if tested. Across conditions, it appears that the rate of decay for task relevant and irrelevant features is similar, though it would appear that task-relevant features do decay somewhat faster.

Our data suggests that the task-relevant advantage develops somewhere between when subjects describe the scene and when they finish reading the change detection instructions. The advantage might be present immediately when the individual objects are first encoded in working memory, though the lack of task effects in Experiment 1 provides tentative evidence against this. Alternatively, the advantage could occur via (relatively) task-sensitive transfer to a longer-term memory. Perhaps the initial accessibility of relevant and irrelevant details is similar, but by 46 s later, task irrelevant features are less strongly encoded.

### Conclusions

Even though subjects knew a memory test was imminent, their performance was still modulated by the demands of the describing task. People cannot encode all the features of an object with equal strength merely by knowing that such maximal encoding would be useful. General instructions like “remember everything you can” appear to have at most a minimal effect on how much (or what types) of visual details are encoded in long-term memory. However, what if subjects knew exactly what features were useful to encode for the change detection task? Would the describing task still influence their memory?

## Experiment 4: Delayed memory test after change detection training

Experiments 2 and 3 tested extended retention intervals and found task relevant encoding advantages. In both of these experiments subjects did not know what sort of features would change, or how their memory would be tested. Perhaps the task-relevant advantage was found because, without explicit guidance, the strength of visual memories was governed by the level of processing of the different features necessary for the describing task. If a person has explicit long-term goals, however, such as memorizing the details likely to change, it may be possible to reallocate memory to the features which are most useful for these long-term goals. Alternatively, it may be that the describing task necessarily influences the strength of long-term memory for the task-relevant features. Experiment 4 tests this by having subjects practice the change detection task 24 times before memory accuracy is measured over extended intervals. This practice should be sufficient to learn what details are most useful to encode, and allow us to test if such knowledge can override the effects of the describing task. By adding practice trials, however, we also introduce new variation in the delay between the describing task and the change detection task. Previously subjects were delayed by reading instructions for the change detection task after finishing the describing task, but now subjects first read those instructions during the practice trials. We expected subjects to speed up when reading the instructions again during the experimental trials; we ran one version of the experiment which allowed this (Experiment 4a), and another which forced a delay of 47 s between describing the last scene and starting change detection (Experiment 4b).

### Method

#### Subjects

Eighty eight college students participated in this experiment, all of whom were new subjects (44 were run in Experiment 4a, and 44 in Experiment 4b).

#### Stimuli

Experimental stimuli were the same as in Experiments 2 and 3. The training stimuli were taken from Experiment 1, with all translation changes removed, for a total of 24 practice scenes.

#### Procedure

Subjects first practiced the paradigm from Experiment 1, where change detection followed immediately after describing a scene. We used ten images that included many obvious changes, selected to help subjects learn what changes to expect. Then subjects practiced the procedure from Experiment 3 on another set of 14 images. Before beginning the final procedure, subjects rested for 30 s to mitigate any fatigue caused by the 24 practice trials. Then they completed an exact replication of Experiment 3, which included reading all the instructions again to preserve timing between tasks. In Experiment 4a subjects started the change detection task immediately after reading the instructions, whereas in Experiment 4b subjects also read the instructions, but were not allowed to proceed to the next task until a total of 47 s had passed. These subjects were to sit quietly and wait during any remaining time between finishing reading the instructions and the end of the 47 s delay. We also used the same set of 22 random orderings of the experimental stimuli as in Experiment 3.

### Results and discussion

#### Timing

In Experiment 4a, subjects spent 16 s per image on the describing task, 30 s reading the instructions for change detection, and then 7 s per image looking for changes. The only difference for Experiment 4b was that subjects were forced to spend 47 s looking at the change detection instructions. Differences in timing between color and naming tasks were minimal for Experiments 4a and 4b. Subjects in the color task were faster by 9% on describing trials, and 6% faster on change detection trials.

#### Task effects

**Figure 8.**
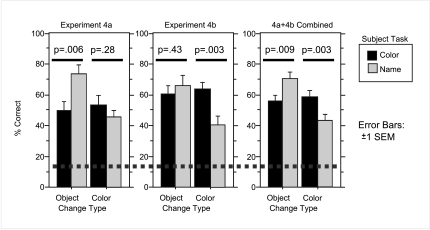
Performance on change detection task in Experiments 4a, 4a, and 4a+4b combined. Dashed line represents chance performance.

Both Experiments 4a and 4b found significant effects of the describing task on change detection rate, though the magnitude of the effect was not consistent across conditions (see Figure 8). In Experiment 4a object changes were significantly easier to detect when subjects had completed the name task (*U* = 127, *p* < .006), whereas the color changes were not significantly affected by task (*U* = 196, *p* < .28). Though Experiment 4b showed the same direction of effects, the pattern of significance was reversed; statistically reliable task effects were found for color changes (*U* =115, *p* < .003), but not for object changes (*U* = 210, *p* < .43). This difference between Experiments 4a and 4b is somewhat surprising given the difference between the two is only how long the delay was between the describing task and the change detection task. Since different groups of subjects were run for Experiments 4a and 4b, it seems likely that much of the difference has to do with inter-subject variability, and the difference in delay may only contribute negligibly. Since the main question of Experiment 4 is the impact of training on task effects, the key point is that for both Experiments 4a and 4b, task effects were found, in the same direction as in Experiments 2 and 3. Since the delay differences were rather small (30 vs. 47 s), and not related to the theoretical question of interest, we conducted one final analysis where Experiments 4a and 4b results were combined to average out any differences. In this final analysis, we still found task effects, this time for both types of change (color *U* = 621, *p* < .003; object *U* = 659, *p* < .009).

### Conclusions

Experiment 4 shows that memory for the features of an object is modulated by a task, such as describing a scene, even when an expected memory test also places clear demands on what features should be remembered best. Though the magnitude of the effect is not as consistent across change types as in Experiments 2 and 3, the fact that a statistically significant effect was found in both Experiments 4a and 4b suggests that task effects on memory will generally be found whenever memory is tested over a significant delay. The reason that Experiment 1 did not find an effect of the describing task appears largely due to the short delay between initial exposure to the scenes and the change detection test.

## Overall conclusion and summary

In this research we investigated how the simple task of describing aspects of objects influences the representation of objects in memory. Short-term memory did not appear to be influenced by the describing task. Longer-term memory, however, was influenced by the describing task, whether subjects knew they were in a memory test or not. Even when subjects had a chance to learn exactly what kind of details to memorize about a scene, the describing task still influenced long-term memory for the scenes.

It is possible that short-term memory would also be influenced by the describing task if the memory test was unexpected. This is a difficult hypothesis to test, however, since at most each subject would only be able to participate in one trial. Furthermore, the need for change-detection instructions presents a lower bound on testing short-term memory. In our current experiments reading and understanding these instructions for the first time typically takes at least 40 s. A simpler change-detection paradigm might reduce this time significantly, but even so the subject would be distracted by reading the instructions. Thus, extending the current task to a short-term surprise paradigm would be exceedingly difficult. In our prior work conducted in VR, however, we have found evidence for task-related modulation of the features in short-term memory (Triesch et al., 2003; Droll et al., 2005), leaving open the possibility of similar effects for natural scenes, if an experiment could be designed which overcomes the difficulties described above. One option would be to use an implicit measure of change detection, so that subjects would not have to read or comprehend any instructions between viewing the natural scene and being tested.

One question about the current results is whether the task-relevant memory advantage found is due only to visual memory, or whether some form of verbal memory is contributing to the effect. The argument here is that when viewing a changed scene, and looking at a crosshair, it might be possible to recall the specific word that was uttered previously. This seems unlikely, however, since subjects in Experiment 1 did not receive any benefit from describing the objects over just looking at them. It is unclear why subjects in Experiments 2, 3, and 4 would have better recall of what they said than subjects in Experiment 1. If anything, they should be much worse, since accurate verbal memory is maintained by actively rehearsing, and subjects had no opportunity to do so because they were constantly describing scenes. While there is long term verbal memory for scene descriptions, it appears to be fairly abstract, and not even sufficient to differentiate between having viewed a picture of a scene, or just a verbal description of it (Intraub & Hoffman, 1992).

Our results extend Hollingworth’s (2004) finding that memory for natural scenes is quite good, even over significant delays. Our work demonstrates detailed, long-lasting memory for the identity of objects in a scene and their color. We also show that this memory is accurate enough to allow subjects to pick out the changed objects from among seven distracters, as opposed to Hollingworth’s task where only one target was highlighted in a 2AFC change/no change task.

An important question is what sort of memory supports this ability. The lack of order effects in Experiment 1 shows that subjects were not using working or short-term memory to detect changes. It is probably unwise, however, to place these memories in the same category as long term memories which last days or years. In Experiment 3, where retention was measured over the length of the experiment, color memory was close to chance by the end of the experiment, even when color was task-relevant. This suggests that color information lasts several minutes, but not longer. Clearly, people can form long-term color memories that last for days or lifetimes, but our task did not lead to this. Melcher (2001) has suggested the existence of a “medium term” visual memory, which lasts over the period of a few minutes, which matches the timecourse we observed.

On the other hand, detection of object additions was still quite good by the end of Experiment 3, though accuracy here too decreased with time. This suggests that the representation of the items in a scene has relatively different temporal dynamics than the representation of surface characteristics of those items. To fully flesh this out, more kinds of surface changes should be explored. It is interesting to note that in Hollingworth (2004), subjects were also quite good at detecting when an object was exchanged for a different exemplar of the same category (e.g., one brand of hammer for another), even after several minutes delay. Clearly, subjects are remembering more than just the category of the objects in the scene; our data suggests not their color, but perhaps some other properties such as shape and local texture. This is further evidence for a medium-term memory, since long-term memory is often thought to be more categorical in nature (Pani, 2000).

Whether it is truly necessary to posit a medium-term visual memory system is unclear, however. Perhaps the behavior observed is due to different dynamics within a single long-term memory system; this is a question for future work. Another question of interest is whether subjects were using explicit memory (that is to say, they could identify explicitly what changed about the object they clicked on), or if their performance was more driven by implicit processes, with most of their correct responses driven by correctly “guessing” where the change occurred, without precisely knowing what the change was. We did not collect any data that can address this question, but it would be interesting in future work to address this question either in a questionnaire at the end of the experiment, or by requiring that subjects also report the nature of the change on each trial.

Our findings could be used to argue against the minimal memory capacity explanation of change blindness. On the other hand, over short intervals, such as in Experiment 1, subjects only detected 60% of color or whole object changes, supporting the argument that the representation of natural scenes is relatively sparse. One explanation for this conflict is that there is a per-scene capacity limit. Only a few objects and features can be accurately encoded and maintained for a single scene, but when multiple scenes must be remembered, the total number of objects maintainable increases. Alternatively, the poor performance for short intervals might be due to a failure to compare the changed item to the memory trace of its pre-changed status, as argued by Simons, Chabris, Schnur, and Levin (2002); Angelone, Levin, and Simons (2003); Hollingworth (2003); Mitroff, Simons, and Levin (2004); as well as by Simons and Rensink (2005). This is likely to be a small contribution in our paradigm, however, since the eight cued objects always included the changed object, and subjects had up to 2 s per cued item to compare it to the memory trace of its pre-change version.

We also found that this accurate memory is formed even when people make no conscious effort to memorize a scene. When we compared performance in Experiments 2 and 3, we found that trying to memorize the scene without any idea of what aspects needed to be memorized did not improve memory much over the encoding caused as a byproduct of engaging in the describing task. This suggests that whenever a task causes a person to attend to aspects of a scene, those aspects are likely to be encoded into a long-term store. Asking a person to memorize a scene is just a different task which causes them to attend to the scene, but does not appear to produce a fundamental difference in how memory is allocated, unless additional guidance is provided.

Furthermore, tasks, which might be considered distracting, might actually elevate the strength of long-term memory beyond what is found when no task is given. Experiment 4 showed that even when subjects know exactly what aspects of a scene need to be encoded for optimal performance on the memory test, a concurrent task such as describing the scene can improve performance when the demands of that task are compatible with the memory test. Thus, in everyday life, where people are constantly engaged in tasks, memory may be significantly better than observed in laboratory experiments where subjects are simply required to remember as much as possible. Whether this improved memory for specific features necessitates a decreased memory for the task-irrelevant features is a question for future research.

Similar issues have been considered in the verbal and word memory literature for some time, though there the question is which words are recalled, not what word features. Tulving and Thomson (1973) suggest that even for simple lists of words, the experimental context influences how words are encoded, and thus, what words are most likely to be recalled at test. Another highly related concept from this literature is transfer-appropriate processing. Morris, Bransford, and Franks (1977) showed that the ability to recall a word was increased when subjects memorized the word using a rhyming task, and then were prompted to recall the word using a rhyming cue, as compared to when a rhyming-unrelated task was performed during memorization. This shows that memory performance is best when the type of processing at test is maximally similar to the type of processing during memorization. Our results can be taken as a general confirmation that a similar kind of effect is seen for visual memories. Our result differs not just in modality, however, but also in that our cue is the changed object itself, whereas in the Morris et al. work, the cue is an entirely different word, who’s connection to the original word has been enhanced by the task. In the domain of memory for sentences and paragraphs, it has also been shown that different types of encoding tasks change what is best remembered, suggesting that memory is anything but a passive store, and that organization and encoding depend on how the information is acquired (Einstein, McDaniel, Owen, & Cote, 1990). This is, however, perhaps less surprising for semantically meaningful information, such as text, than it is for visual memory for the features of objects in natural scenes.

We found that whole object changes were detected more frequently than color changes in Experiments 2, 3, and 4. This may suggest that long-term visual memory is organized into individual objects, with different features bound to each object. Thus, if you can detect a color change, you should be able to detect a whole object change, since in order to encode the color of the object; you would also need to encode the object’s identity. Conversely, our results suggest that all the features of an object need not be equally easy to recall, so being able to detect an object change does not necessarily mean that a color change could be detected.

While this seems like a sensible organization of visual memory, it is possible to imagine how to design an experiment where the reverse set of results might be found. Simply make the object changes very subtle, such as changing the object type from a tube of toothpaste to a tube of paint, while making very large color changes (such as a white tube of toothpaste that becomes Day-Glo orange). Thus, while our results are compatible with features being bound into individual objects, further research will be necessary to show the extent to which this is the actual organization of visual memory.

All of the task effects we found suggest that different features of objects are not equally easy to recall. Instead, the ease of recalling a feature depends, at least in part, on what the subject was doing when they viewed the object. This suggests that visual memory (be it long- or medium-term) is not allocated equally across the features of an object. Rather, different features are encoded at different strengths, depending on the task(s) that caused them to be encoded in the first place. As discussed in the introduction, this interpretation of the results is compatible with previous experiments with more artificial scenes, such as our own work in Virtual Reality.

There are other possible explanations of our current results, however. In particular, encoding could be the same, irrespective of task, but there could be a difference in what happens at recall; for instance, the naming task could have cued subjects to attend to object identity during the recall stage, increasing the likelihood of detecting object changes. This could be tested in future experiments by having subjects complete two blocks of trials: one with the naming task, and one with the color task, before having their memory tested. At test time, subjects would be shown images from both blocks, intermixed in random order. If the describing task influences encoding, then its effects should still be measurable even when images from the two blocks are intermixed at recall. On the other hand, if the describing task just biases recall, then doing both types of tasks before the recall stage should significantly reduce or eliminate task effects. This should be a particularly interesting area for future research.

## Footnotes

1 While our working hypothesis is that the strength of visual memory traces can vary, since we can only test people’s recall ability, it is possible that some other mechanism is at play. For instance, recall performance might be better because of several different reasons, including increased encoding strength of the memory trace, less interference, or because the task-relevance of the feature increases the ease with which the trace of that feature can be recalled during the memory test. The purpose of this research is to show that memory performance depends on the task; future work will be necessary to confirm that it is due to differences in encoding strength.

2 Note that for conciseness, when the term memory is used in this paper, we are referring to visual memory, as opposed to verbal or other modal memory systems.

3 It is possible that chance performance was actually higher than 12.5%. We did not conduct a no-change version of the experiment to see if subjects could guess what the changed items would be without having seen both versions of each scene. It seems unlikely that subjects would be good at this, however, as the most likely cue for such guesses, photo manipulation artifacts, were only visible during the describing part of the experiment, when subjects had no reason to look for them, and furthermore, these artifacts were rather subtle. Therefore, 12.5% seems a good estimate of chance performance.
